# Baseline malaria prevalence and care-seeking behaviours in rural Madagascar prior to a trial to expand malaria community case management to all ages

**DOI:** 10.1186/s12936-021-03956-z

**Published:** 2021-10-26

**Authors:** Dean Sayre, Laura C. Steinhardt, Judickaelle Irinantenaina, Catherine Dentinger, Tsinjo Fehizoro Rasoanaivo, Laurent Kapesa, Jocelyn Razafindrakoto, Agathe Legrand, Nicole Prada, Julie Gutman, Lauren Lewis, Reziky Tiandraza Mangahasimbola, Mauricette Andriamananjara, Aimée Vololoniala Ravaoarinosy, Nicolas Ralemary, Andres Garchitorena, Aina Harimanana

**Affiliations:** 1 Epidemic Intelligence Service, Centers for Disease Control and Prevention, 1600 Clifton Rd, Atlanta, GA USA; 2 U.S. President’s Malaria Initiative, Centers for Disease Control and Prevention, 1600 Clifton Rd, Atlanta, GA USA; 3 Malaria Branch, Division of Parasitic Diseases and Malaria, Center for Global Health, CDC Atlanta, 1600 Clifton Rd, Atlanta, GA USA; 4 U.S. President’s Malaria Initiative, Centers for Disease Control and Prevention, Lot 207A, Point Liberty, Andranoro, Antehiroka, 105 Antananarivo, Madagascar; 5 U.S. President’s Malaria Initiative, USAID, Lot 207A, Point Liberty, Andranoro, Antehiroka, 105 Antananarivo, Madagascar; 6 Institut Pasteur de Madagascar , BP 1274 Ambatofotsikely, 101 Antananarivo, Madagascar; 7 MIVEGEC, Univ. Montpellier, CNRS, IRD, 911 Av Agropolis, Montpellier, France; 8 National Malaria Control Programme, Ministry of Health, Antananarivo, Madagascar; 9 Ministry of Public Health, Farafangana District Office, Farafangana, Madagascar

## Abstract

**Background:**

Integrated community case management of malaria, pneumonia, and diarrhoea can reduce mortality in children under five years (CU5) in resource-poor countries. There is growing interest in expanding malaria community case management (mCCM) to older individuals, but limited empirical evidence exists to guide this expansion. As part of a two-year cluster-randomized trial of mCCM expansion to all ages in southeastern Madagascar, a cross-sectional survey was conducted to assess baseline malaria prevalence and healthcare-seeking behaviours.

**Methods:**

Two enumeration areas (EAs) were randomly chosen from each catchment area of the 30 health facilities (HFs) in Farafangana district designated for the mCCM age expansion trial; 28 households were randomly selected from each EA for the survey. All household members were asked about recent illness and care-seeking, and malaria prevalence was assessed by rapid diagnostic test (RDT) among children < 15 years of age. Weighted population estimates and Rao-Scott chi-squared tests were used to examine illness, care-seeking, malaria case management, and malaria prevalence patterns.

**Results:**

Illness in the two weeks prior to the survey was reported by 459 (6.7%) of 8050 respondents in 334 of 1458 households surveyed. Most individuals noting illness (375/459; 82.3%) reported fever. Of those reporting fever, 28.7% (112/375) sought care; this did not vary by participant age (p = 0.66). Most participants seeking care for fever visited public HFs (48/112, 46.8%), or community healthcare volunteers (CHVs) (40/112, 31.0%). Of those presenting with fever at HFs or to CHVs, 87.0% and 71.0%, respectively, reported being tested for malaria. RDT positivity among 3,316 tested children < 15 years was 25.4% (CI: 21.5–29.4%) and increased with age: 16.9% in CU5 versus 31.8% in 5–14-year-olds (p < 0.0001). Among RDT-positive individuals, 28.4% of CU5 and 18.5% of 5–14-year-olds reported fever in the two weeks prior to survey (p = 0.044).

**Conclusions:**

The higher prevalence of malaria among older individuals coupled with high rates of malaria testing for those who sought care at CHVs suggest that expanding mCCM to older individuals may substantially increase the number of infected individuals with improved access to care, which could have additional favorable effects on malaria transmission.

**Supplementary Information:**

The online version contains supplementary material available at 10.1186/s12936-021-03956-z.

## Background

Integrated community case management (iCCM) is a strategy that provides community healthcare workers/volunteers (CHVs) with training and resources required to diagnose and treat children under 5 years (CU5) with uncomplicated malaria, diarrhoea, and pneumonia in community settings. These three diseases are among the most common causes of childhood mortality in resource-poor settings [1] and iCCM expands access to potentially life-saving care, especially for those who have challenges accessing health facilities (HF) [1]. Multiple studies have demonstrated both improvements in both care-seeking for febrile illness and mortality reductions in CU5 following the introduction of iCCM to a population [2–4]. The success of iCCM in CU5, coupled with data demonstrating the association between delayed malaria care and progression to severe disease [5] has led to increasing interest in expanding access for malaria community case management (mCCM) to older individuals; however, there is a paucity of evidence that demonstrates the feasibility and effectiveness of expanding access to treatment to all ages. Rwanda officially began offering mCCM to all ages in 2015 and is one of the few countries that have conducted and published an assessment of the impacts of this change. Investigators in Rwanda conducted a retrospective review of health facility data that suggested broad public acceptability for expanding CCM-malaria to all age groups [6]. However, this study was limited to retrospective analysis of routine data from a non-representative subset of the population and failed to show any associated difference in malaria burden or care-seeking behaviours [6]. More evidence is needed on the feasibility and potential impacts of such an approach in order to inform policies and implementation in other countries.

Madagascar is among several countries in sub-Saharan Africa considering expanding access to mCCM for older populations to address recent increases in malaria transmission rates and persistently low rates of care-seeking behaviour. Malaria prevalence in Madagascar is markedly heterogeneous, but has increased overall since 2010 as evidenced by both routine surveillance data and analyses of data from several Malaria Indicator Surveys (MIS) [7–10]. The World Health Organization (WHO) estimates malaria incidence to have increased from 42 cases per 1000 individuals in 2010 to 76 cases per 1000 people in 2019 [10]. Estimates of malaria-attributable deaths showed an analogous increase during the same time by WHO estimations, from 10 to 18 per 100,000 population [10]. Based on data from the 2016 MIS, an estimated 46% of CU5 with febrile illness were seen by a provider for care/advice [7]. Only 15.5% of CU5 with fever had blood taken for a diagnostic malaria test [7], despite national guidelines recommending that all individuals suspected to have malaria be tested.

Madagascar’s current National Strategic Plan 2018–2022 outlines the policies adopted to promote effective malaria case management as part of a larger plan attempting decrease the national disease burden. Included in this plan is the expansion of access to mCCM to older individuals [11], but this strategy has yet to be implemented. Under current national policies, CHVs are responsible for providing iCCM for CU5, malnutrition screening and health education, among other activities. Two CHVs are appointed by the community in every fokontany (smallest administrative level). CHVs do not receive a formal salary but may earn money from the sale of medicines for diarrhea and pneumonia as part of iCCM. To aid in determining the effectiveness of expanding mCCM to all ages, in 2019 a cluster-randomized trial comparing traditional mCCM for CU5 to a mCCM strategy expanded to all ages was initiated in the rural district of Farafangana, along the southeast coast of Madagascar. Here, malaria prevalence and routine healthcare-seeking behaviour data collected from the study population during a baseline cross-sectional survey are described.

## Methods

### Study setting

Farafangana is a coastal district in southeastern Madagascar. It has a largely rural population of nearly 500,000 individuals served by 38 public health facilities and over 600 CHVs. Geographic accessibility to primary care is low in the district, with less than 40% of the population living within 5 km from the nearest health facility. Malaria transmission in the district is seasonally variable with a period of increased transmission from roughly October to April [9, 12]. Passive surveillance estimates from 2015 to 2017 indicated an average annual incidence of just under 100 cases per 1000 population, and the 2016 MIS indicated a prevalence of 9.0% in CU5 in this region of the country [7].

### Survey sampling

All public HFs and their surrounding catchment areas in the study district were delineated and assessed for accessibility, security and implementation of iCCM (see Additional file 1). Of the 38 HFs present, those in areas with known limited accessibility and/or safety concerns were excluded, as were those in the city of Farafangana because iCCM is not performed in urban areas in Madagascar (CHVs refer patients to HFs). Thirty HFs and their corresponding catchment areas were selected for study inclusion based on those criteria. Each catchment area was subsequently split into 2-km by 2-km enumeration areas (EAs). Two EAs within each catchment area were chosen for baseline survey inclusion by simple random selection. All structures visible by satellite imagery within each selected EA were included in a GIS database and integrated into the survey teams’ tablets via the OsmAnd application (Amstelveen, The Netherlands, https://osmand.net/). This served as a guide for complete household enumeration and sample population census in the field. If a selected area had fewer than 60 structures visible by satellite imagery, one of the adjacent enumeration areas was selected at random and the two EAs were joined to create a larger area for sampling. If the resulting area still had fewer than 60 structures, the process was repeated until this threshold was achieved. The resulting merged areas were treated as a single EA for sampling purposes. Survey teams mapped the selected EAs to list all occupied dwellings and their inhabitants. From the list of occupied households, 28 per EA were selected by simple random sampling for participation in the baseline survey, for a total of 1680 eligible households. Eligible households that refused participation or could not be contacted for survey enrollment were not replaced. Sample size was calculated to provide adequate power for interventional trial; see Additional file 2 for details.

### Household surveys

Household-level data, including distance to CHV/HF, perceptions of CHV/HFs, most recent visit to CHV/HF by any household member, ownership of assets, and a household listing of members, were solicited from the head of the household or a usual adult household member. Data on recent illnesses and subsequent care-seeking were collected on all individuals living within participating households. Survey data reflect responses obtained from the self-identifying head of household for all household members or another adult household member when the head of household was unavailable. Surveys were conducted using digital tablets loaded with REDCap software version v8.5.17 (Vanderbilt University, https://www.project-redcap.org/).

All household members $\ge$ 2 months and $<$ 15 years of age were offered a rapid malaria diagnostic test (RDT) capable of detecting both *Plasmodium falciparum* and other *Plasmodium* species (SD Bioline Malaria Ag P.f/Pan, Abbott Laboratories, Abbott Park, Illinois). Individuals testing positive for malaria without a history of receiving anti-malarial medications within 28 days prior to survey were provided with artesunate-amodiaquine in accordance with Madagascar national guidelines. Additional capillary blood samples from consenting individuals in this age range were collected on Whatman 903 protein saver cards (Sigma-Aldrich, MilliporeSigma, St. Louis, MO, USA) for future laboratory analyses.

### Data analysis

Data were analysed using R version 4.0.2 (R Foundation for Statistical Computing, Vienna, Austria). The survey package version 4.0 was used to account for cluster design effects and survey weights in statistical analyses and population estimates. All percentages given are weighted population estimates unless otherwise noted; all reported confidence intervals are 95% confidence intervals (CI). Chi-squared tests with the Rao and Scott correction [13] were used for categorical data, and Kruskal–Wallis tests were used to assess for differences in nonparametric numeric data across several groups.

Geospatial malaria prevalence surfaces were created by assigning the weighted prevalence of malaria RDT positivity within each EA to its geographic centroid and interpolating prevalence throughout the study area using the ordinary kriging predictor.

## Results

Data were collected from 8050 individuals representing 1458 distinct households in Farafangana from October to December 2019. These households represent 91.5% of eligible households; household participation ranged from 60.7% to 100% among EAs. Measured socioeconomic status (SES) throughout the survey population was fairly homogeneous, with surrogate markers, such as head of household education and structural characteristics of the home skewed toward low SES (Table 1).

**Table 1:** Characteristics of households visited during cross sectional survey in Farafangana, Madagascar—2019

		Mean (years)	95% CI	Observed
Head of house age		41.6	(40, 43.2)	920

		Percent (%)	95% CI	Observed
Head of house gender	Male	57.9	(51.7, 64.1)	572
	Female	42.1	(35.9, 48.3)	347
Head of house education	Did not finish primary school	84.7	(81.1, 88.3)	1248
	Finished primary school, or beyond	15.3	(11.7, 18.9)	209
Head of house occupation	Farmer	62.6	(54, 71.2)	902
	Day laborer	11.7	(8.8, 14.6)	200
	Other	25.7	(18.6, 32.9)	355
Household floor material^a^	Primitive	86.0	(80.7, 91.4)	1269
	Non-primitive	4.2	(2.7, 5.6)	64
	Other	9.8	(4.4, 15.2)	124
Household roof material^b^	Primitive	75.1	(68.5, 81.7)	1212
	Non-primitive	12.0	(9.5, 14.5)	163
	Other	12.9	(7.9, 17.9)	162
Household toilet^c^	None/nature	68.0	(62.5, 73.4)	951
	Primitive	31.7	(26.1, 37.3)	503
	Non-primitive	0.1	(0, 0.4)	1
	Other	0.2	(0, 0.5)	2
Electricity in the home	No	94.5	(92.3, 96.7)	1364
	Yes	5.5	(3.3, 7.7)	93

		Mean	95% CI	Observed
Nets in household	Per sleeping space	0.97	(0.95, 1)	1404
	Used per sleeping space	0.92	(0.91, 0.93)	1403
	Per individual	0.46	(0.44, 0.48)	1453

Weighted population average responses are reported along with 95% confidence intervals. Raw counts observed in the survey are given in the last column

^a^Categorization of household floor materials: Primitive—“Dirt”, “Dung”, “Wooden boards”, “Palm/Bamboo”. Non-primitive—“Parquet/waxed wood”, “Vinyl/asphalt”, “Tiles”, “Cement”, “Carpet”

^b^Categorization of household roof materials: Primitive—“Palms”, “Grass”, “Mats”, “Bamboo” “Wooden Boards”. Non-primitive—“Sheet metal”, “Wood”, “Zinc/Cement Fiber”, “Tiles”, “Cement”, “Shingles”

^c^Categorization of household toilet: Primitive—“Self-ventilated improved latrine", “Latrine with washable slab”. “Sodless Latrine/Open Hole”, “Composting Toilet”, “Bucket”, “Hanging Latrine”. Non-primitive—“Flushing toilet regardless of connection”

**Fig. 1:**
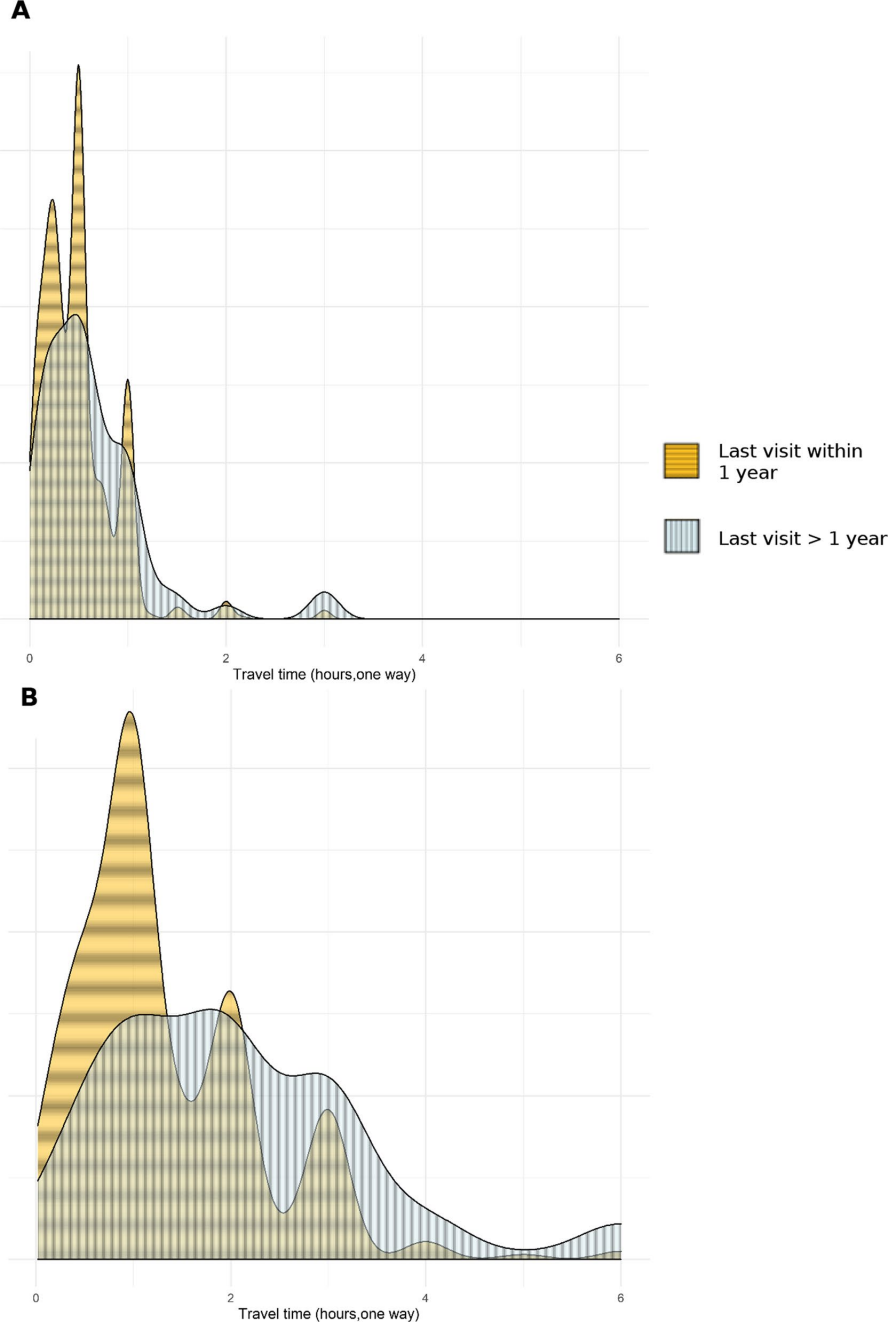
Last visit to healthcare provider within each surveyed household by travel time by to provider in Farafangana, October–December 2019. Density plots showing household travel time to **A** community health volunteers and **B** health facilities, stratified by last visit to each within the household

At least one member of the household had visited a CHV within the past year in 76.1% (95% confidence interval [CI]: 71.0–81.2%) of households throughout the study population (range by EA: 22.2–100% p = 0.045, adjusted Chi-squared test). Household use of HFs within the past year was 81.6% (CI: 77.4–85.7%, range by EA: 28.0–100%, p = 0.102, adjusted Chi-squared, Table 2). At the household level, seeking care for febrile illnesses was the leading cause for the most recent visits to both CHVs (83.3%, CI: 79.8–86.8%) and HFs (52.2%, CI: 47.8–56.6%). Overall satisfaction with care provided by HFs and CHVs was similarly high, with 96.2% (94.5–97.9%) of heads of household “somewhat satisfied” or “very satisfied” with care received at HFs and 96.1% (94.0–98.2%) “somewhat satisfied” or “very satisfied” with care provided by CHVs (Table 2). Mean travel time to HF from the home was 1.60 h (CI: 1.30–1.80 h). Mean travel time from the home to the CHV was 0.48 h (CI: 0.38–0.57 h). Recorded travel time to HF was associated with recency of any household member seeking care there (p = 0.012, Kruskal–Wallis test, Fig. 1). A similar association between care-seeking at the CHV and required travel time failed to reach statistical significance (p = 0.073, Kruskal–Wallis test).

**Table 2:** Household characteristics related to healthcare providers—Farafangana, 2019

		Mean	95% CI	Observed
Community health volunteers (CHVs)				
Time to provider (hours, one-way)		0.48	(0.38, 0.57)	1272

		Percent (%)	95% CI	Observed
Last visit—any member	1 year or less	76.1	(71, 81.2)	1096
	> 1 year	23.9	(18.8, 29)	334
Reason for last visit—any member	Fever	83.3	(79.8, 86.8)	1011
	Other	16.7	(13.2, 20.2)	237
Satisfaction	Not satisfied	3.8	(2.1, 5.5)	48
	Satisfied	96.2	(94.5, 97.9)	1201

		Mean	95% CI	Observed
Health facilities (HFs)				
Time to provider (hours, one-way)		1.6	(1.3, 1.8)	1332

		Percent (%)	95% CI	Observed
Last visit—any member	1 year or less	81.6	(77.4, 85.7)	1156
	> 1 year	18.4	(14.3, 22.6)	275
Reason for last visit—any member	Fever	52.2	(47.8, 56.6)	691
	Other	47.8	(43.4, 52.2)	665
Satisfaction	Not satisfied	3.9	(1.8, 6)	42
	Satisfied	96.1	(94, 98.2)	1315

Weighted population average responses are reported along with 95% confidence intervals. Raw counts observed in the survey are given in the last column

Individuals under five years made up 22.7% (CI: 20.9–24.4%) of the study population, with another 28.7% aged 5–14 years (CI: 26.8–30.6%; Table 3). In the two weeks prior to survey, an estimated 5.5% (CI: 4.7–6.3%) of the population noted experiencing a febrile illness, 28.7% (CI: 22.2–35.3%) of whom sought medical advice/care. Among those seeking care for febrile illness, 74.3% (CI: 64.7–83.9%) reported having a test for malaria as part of their diagnostic work-up (Table 3). Self-reported febrile illness within the previous two weeks varied by age. Incidence of febrile illness among CU5 was estimated at 8.2% (CI: 6.2–10.7), 29.9% (CI: 20.4–41.4%) of whom sought medical care or advice outside the home, and 23.4% (CI: 14.4–35.8%) of whom received a diagnostic test for malaria. Among children 5–14 years, incidence of febrile illness was estimated to be 7.5% (CI: 6.0–9.3), 28.5% (CI: 18.8–40.8%) of whom sought medical attention and 20.9% (CI: 13.1–31.6%) of whom were tested for malaria infection. An estimated 3.1% (CI: 2.4–3.9%) of individuals 15 years and older had febrile illness in the two weeks prior to the interview. Of these individuals, 27.7% (CI: 16.4–42.9%) sought medical attention for this illness and 19.6% (CI: 10.2–34.3%) were tested for malaria infection as part of their diagnostic workup (see Additional file 3). Neither differences in incidence of febrile illness nor malaria testing by enumeration area were statistically significant (p = 0.66 and 0.26, respectively, adjusted Chi-squared test). However, a significant association between care-seeking for febrile illnesses and EA was observed (range: 0–100%, p = 0.0015, adjusted Chi-squared test).

**Table 3:** Individuals’ characteristics, history of illness, and care-seeking behaviour during cross sectional survey in Farafangana, Madagascar, October–December, 2019

	Percent (%)	95% CI	Observed
Male	52.4	(51.5, 53.3)	4162
Female	47.6	(46.7, 48.5)	3888
Under 5	22.7	(20.9, 24.4)	1799
5–14 years	28.7	(26.8, 30.6)	2345
15 + years	48.6	(47.1, 50.2)	3906
Not ill in past 2 weeks	93.3	(92.3, 94.3)	7591
Ill in past 2 weeks	6.7	(5.7, 7.7)	459
Among ill			
No fever	17.6	(12.2, 22.9)	82/459
Fever	82.3	(76.9, 87.6)	375/459
Unknown	0.2	(0, 0.4)	2/459
Among febrile			
No care	71.3	(64.7, 77.8)	258/375
Any care	28.7	(22.2, 35.3)	112/375
Care at HF and/or CHV	23.0	(17.0, 29.0)	88/375
Among care-seeking			
Not tested	25.7	(16.1, 35.3)	30/112
Tested	74.3	(64.7, 83.9)	82/112

Weighted population average responses are reported along with 95% confidence intervals. Raw counts observed in the survey are given in the last column

**Fig. 2:**
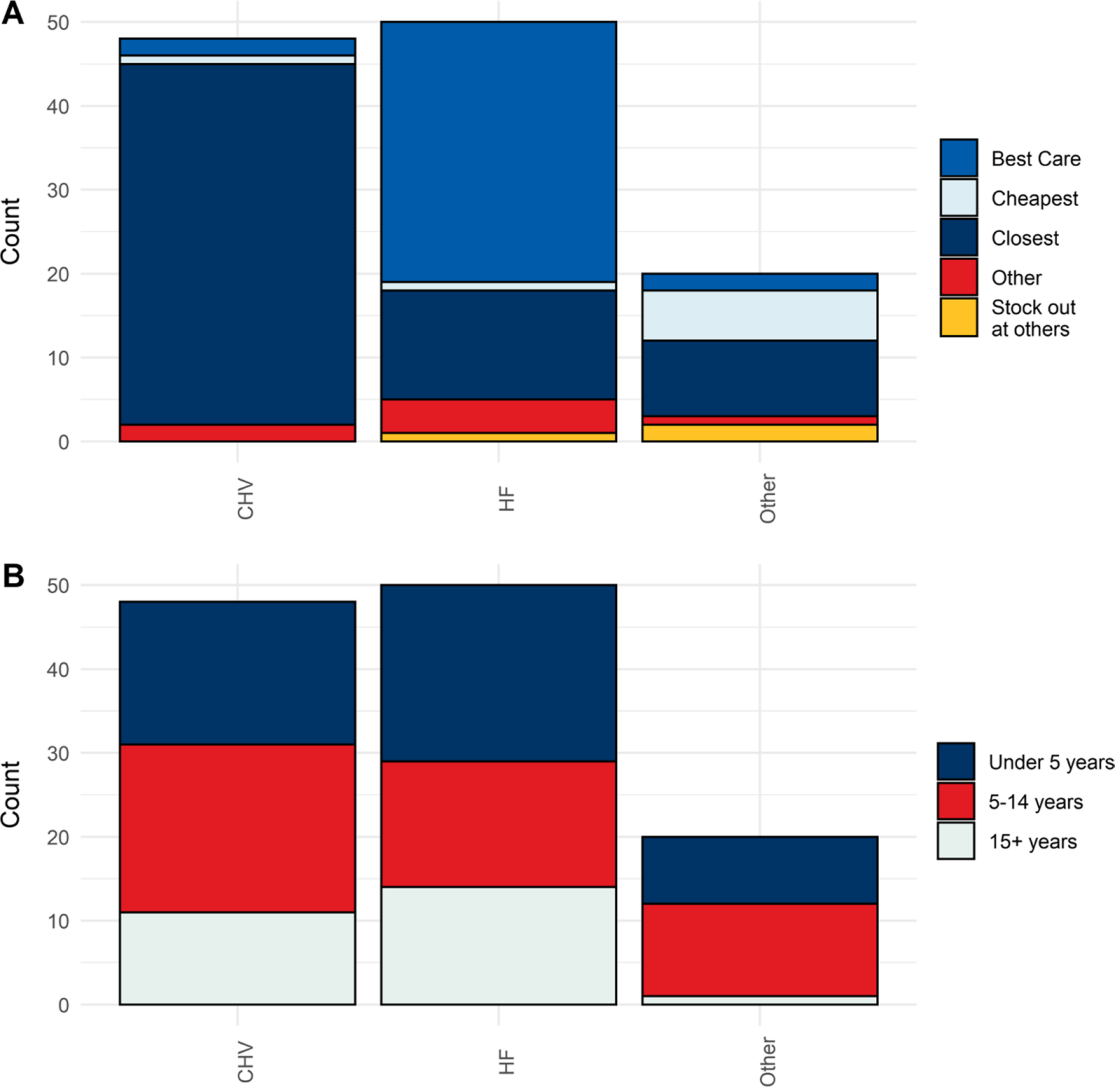
Type of provider visited by those seeking care for febrile illness in Farafangana, October–December, 2019.** A** Raw counts of individuals seeking care for febrile illnesses occurring within the two weeks prior to survey, grouped by type of provider(CHV – community health volunteer, HF – public health facility, Other^1^). Colors represent each individual’s stated primary reason for choosing a provider. ***B.*** Raw counts of individuals seeking care for febrile illnesses occurring within the two weeks prior to survey, grouped by type of provider. Colors represent each individual’s age. ^1^ ‘Other’ category includes: private health facility, pharmacy, marketplace, self-medication, and neighbors

Public HFs were the most visited sources of care among those who sought care for recent febrile illness (46.8%, CI: 29.2–64.4%), followed by CHVs (31.0%, CI: 19.1–42.8%). Other notable sources of care noted included self-medication (6.6%), private health facilities (4.1%), marketplaces (1.8%), and pharmacies (1.3%). Individuals of all ages reported seeking care at HFs and CHVs: there was no difference in the overall age profile of individuals cared for by these two types of health providers (p = 0.99, Kruskal–Wallis test). A majority (42/47) of those who sought care from the CHVs for fever cited their proximity as the primary reason for choosing this source of care. A majority (29/48) of those who chose to visit a public HF for care noted perceived high quality of care as the primary reason (Fig. 2).

A high percentage of individuals who sought care for febrile illness were tested for malaria at either HFs (87.0%, CI: 77.4–96.7%) and CHVs (71.0%, CI: 52.4–89.6%), with no statistical differences in testing between CHVs and HFs (p = 0.127, see Additional file 4). Most reported consults for febrile illness and most malaria diagnostic tests used by CHVs were used for participants older than five years (29/47 among 5 + years and 18/29 among CU5, respectively). Rates of malaria testing did not significantly differ at either CHVs or HFs by participant age groups (p = 0.278 and 0.165, respectively).

**Fig. 3:**
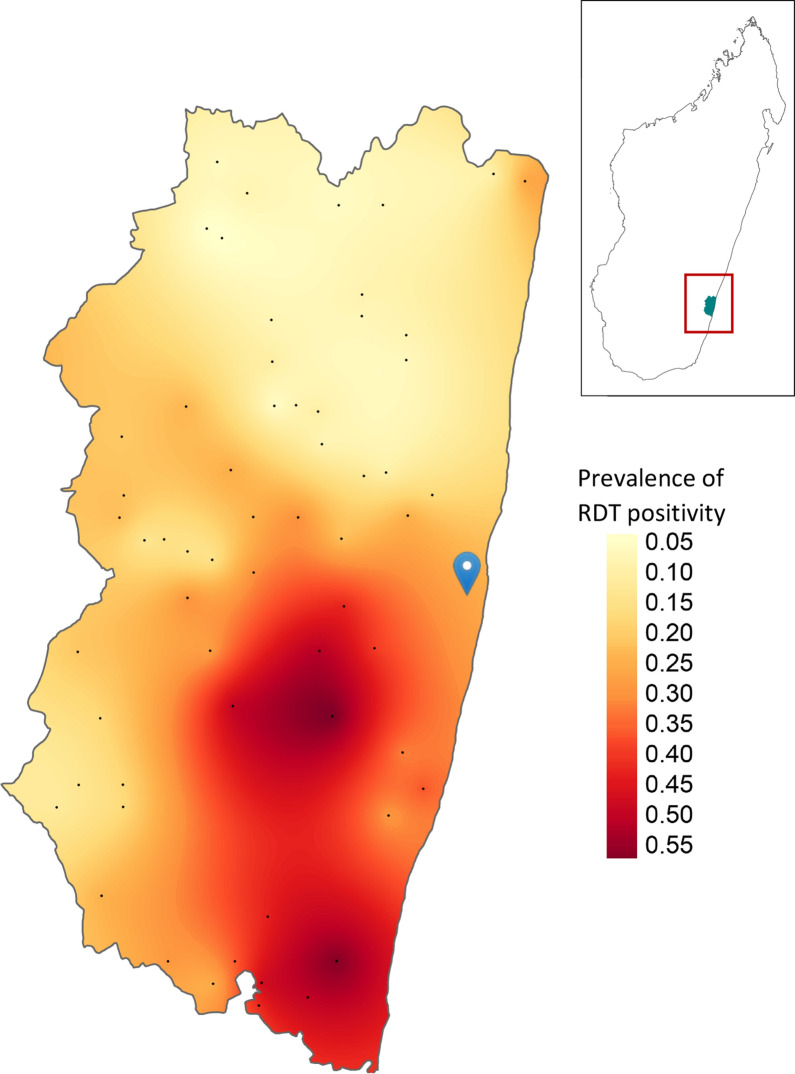
Observed prevalence of malaria RDT positivity among children < 15 years by household screen, Farafangana, Madagascar, October–November 2019. Colors represent interpolated malaria prevalence using ordinary Kriging. Prevalence displayed is RDT positivity for any species of *Plasmodium.* Black points are geographic centroids of areas surveyed. Blue icon represents the location of Farafangana city. Insert shows location of Farafangana in Madagascar. Shapefile from Humanitarian Data Exchange (https://data.humdata.org/)

**Fig. 4:**
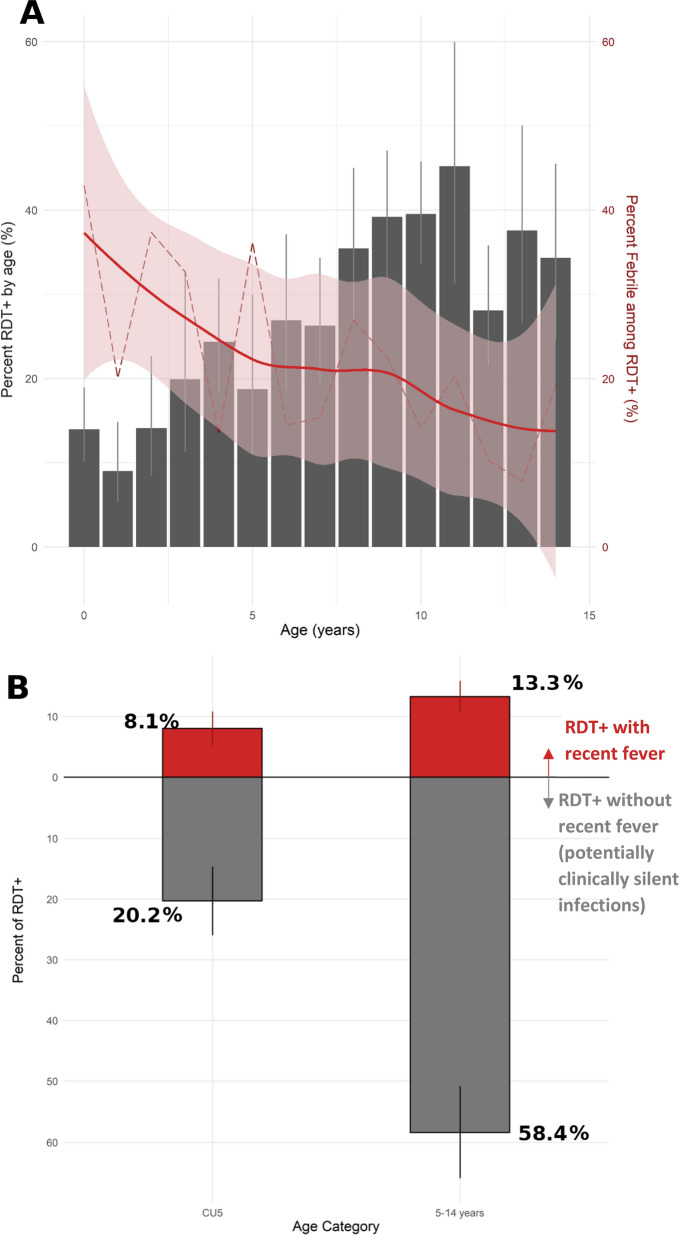
Prevalence of malaria RDT positivity and history of fever among all RDT + children 2 months to 14 years, by age, Farafangana 2019. **A** Dark gray bars show population weighted malaria prevalence (any species) estimates as measured by RDT by age in years (x-axis, rounded down to last complete year). Vertical lines in light gray illustrate 95% CI for RDT + prevalence. Dashed, jagged red line shows weighted population estimates for the percentage of those with preceding fever among all RDT + by age in years. Smooth, solid line illustrates modeled percentage of RDT + individuals with preceding fever by age using LOESS regression. Light red shading surrounding smooth regression line illustrates 95% CI of model. **B** Weighted percentages of all RDT + individuals grouped by age and fever status. Length of red bars extending upward from x-axis represent percentage of RDT + individuals noting fever within two weeks of survey. Length of gray bars extending downward from x-axis represent percentage of RDT + individuals without preceding fever. Vertical lines associated with each bar represent 95% confidence intervals

Malaria RDT prevalence among 3316 children aged between 2 months and 15 years was estimated to be 25.4% (CI: 21.5–29.4%). Prevalence varied significantly by EA (range: 0–63.5%, p < 0.0001, adjusted Chi-squared test, Fig. 3) and age; 16.9% (CI: 12.4–22.8%) of CU5 tested positive whereas 31.8% (CI: 27.3–36.6%) of those aged 5–14 years were positive by RDT (p < 0.0001). The majority of children who were RDT positive did not report having fever in the previous two weeks (78.6%, Table 4), though a larger fraction of cases detected in CU5 had preceding fever (28.4%, CI: 19.7–39.1%) compared with children 5–14 years (18.5%, CI: 14.3–23.7%, p = 0.0437). Of all individuals positive by RDT, 8.1% (CI: 5.3–10.8%) were CU5 with associated fever and 13.3% (CI: 10.7–15.9%) were children 5–14 years with associated fever (Fig. 4). *Plasmodium falciparum* was the infecting species in most individuals with malaria; 99.9% of infections diagnosed as *P. falciparum* mono-species or mixed species including *P. falciparum*.

**Table 4:** Malaria prevalence among children $\le$ 14 years during cross section survey in Farafangana, Madagascar, October–December 2019

	Percent (%)	95% CI	Observed
RDT positive overall	25.4	(21.5, 29.4)	760/3316
Recent fever (previous 2 weeks)	21.3	(16.7, 26)	149/760

	Percent (%)	95% CI	Observed
Gender			
RDT + female	23.6	(18.5, 29.7)	357/760
RDT + male	27.4	(23.8, 31.4)	403/760
RDT + under 5 years	16.9	(12.4, 22.8)	206/760
Recent fever	28.4	(19.7, 39.1)	59/206
Age			
RDT + 5–14 years	31.8	(27.3, 36.6)	554/760
Recent fever	18.5	(14.3, 23.7)	90/554

Weighted population average responses are reported along with 95% confidence intervals. Raw counts observed in the survey are given in the last column

## Discussion

Ensuring universal access to malaria diagnosis and treatment is a key global priority according to WHO to reduce the current burden of malaria [14]. Given the success of iCCM in increasing health care access for common childhood diseases, including malaria, several national malaria programmes are considering expanding mCCM (one of the components of iCCM) to older age groups. Here, baseline conditions in a rural district of Madagascar are documented ahead of a randomized cluster trial to assess the effectiveness of a mCCM expansion to all ages. The results presented convey both promise and potential challenges for the proposed expansion of mCCM to older ages.

Malaria prevalence in children 2 months to 14 years in this study population was higher compared to previously published community survey results [7, 9]. This was in part by design, as a known pocket of higher malaria transmission relative to the rest of the country was targeted and the survey was conducted during the high transmission season. Additionally, documented estimates of malaria prevalence frequently focus on CU5, though higher prevalence in older children have been documented in several countries [15–17], including Madagascar (R Ratovoson, personal communication). Older children thus represent a potential reservoir of malaria transmission, and it follows that an effective expansion of community case management to this portion of the population could reduce both malaria-attributed morbidity and malaria transmission across Madagascar. However, significantly more malaria infections in the 5–14 year age group were asymptomatic, a feature that has been observed elsewhere [15, 18, 19] and could limit the population-level effects of an age expansion of mCCM. Of the infections documented, 21.4% were associated with fever, suggesting that the vast majority of infections in the study area might not be detected by mCCM given the lack of clinical symptoms prompting medical care. While this may have implications for the ability of age-expanded mCCM to alter malaria transmission, such a change in policy could drastically increase the overall eligibility for malaria care among those needing it; there were 1.6 times as many symptomatic malaria infections in children aged 5–14 years compared to those in CU5.

To be fully effective, the availability of mCCM to older age groups must be coupled with an accompanying increase in use by these groups. Previous work has demonstrated that malaria is largely not considered to be a major problem by Madagascar residents [20], with a high percentage of individuals displaying a lack of understanding of the disease [21]. Many individuals only seek care after the onset of symptoms of more serious disease and care-seeking is initially driven by proximity, convenience, and cost, with the formal health facility often viewed as a last resort [20]. CHVs, as local extensions of health facilities, are thus potentially ideally positioned to provide malaria treatment, as they are on average closer to the home. Average one-way travel time to CHVs in our study was roughly one-third that of the average one-way travel time to HFs. Proximity was overwhelmingly the primary reason cited by those choosing to seek care from CHVs, underlining the importance of continued or enhanced access to high-quality health services for malaria testing and treatment from the CHVs for at least a specific subpopulation of individuals. In addition, CHVs were perceived to provide a similarly high level of care compared to HFs, although this did not translate into a high degree of care-seeking among individuals with febrile illness. Only 28.7% of those with recent fever sought medical attention, which is substantially lower than care-seeking estimates previously reported in this region of Madagascar (MIS 2016: 40.4%) [7]. Even among younger individuals, care-seeking rates were strikingly low; rates in younger individuals were not statistically different than those of older individuals.

The ability to modify existing health-seeking behaviours through expansion of mCCM is not well established. There are several examples of improvement of care-seeking rates following the introduction of iCCM to naïve populations where it was previously effectively non-existent [2, 3]. However, recent publications from Malawi [22] and Rwanda [6] underscore the difficulty in increasing care-seeking behaviour through support or expansion of established community case management programs. Potential pre-existing noncompliance with current restrictions on age eligibility for mCCM may present an additional complication in Madagascar. Depending on the extent to which CHVs are currently managing febrile illnesses in older individuals, the official expansion of malaria community case management to older ages in Madagascar may or may not practically increase access to mCCM for a large population once implemented. Additionally, the widespread use of CHVs by individuals other than CU5 may mask the effectiveness of age-expanded mCCM during the ongoing interventional trial. However, by specifically training CHVs on care for individuals older than 5 years and by providing the appropriate anti-malarial treatments for each age group, an official expansion of mCCM to all ages could improve the quality of care for those seeking care at the community level.

Beyond access and use of CHV services, effective mCCM requires an adequate stock of malaria therapeutic and diagnostic commodities and CHV compliance with national guidelines. A number of HFs and CHVs across the country have noted insufficient stocks of needed commodities [23], and reports suggest that providers in at least some regions of Madagascar regularly provide anti-malarial medication in the absence of a positive RDT, due to either RDT stock-outs or disregard of negative test results [23, 24]. This survey was not designed to accurately estimate the degree to which CHVs provided anti-malarial medication in the absence of a positive RDT. However, participant reports suggest that CHV compliance with national guidelines to test all febrile patients for malaria was comparable to that attained in public HFs and was much higher than figures previously reported from private facilities in Madagascar [25].

This study had several limitations. First, the number of participants noting a febrile illness in the two weeks prior to survey was much lower than assumed during sample size calculations. This resulted in lower sample size for our analyses of individual care-seeking behaviours and could explain the lack of statistically significant associations with several covariates. Care should be taken when interpreting non-significant results presented here. Second, as with most local studies, results cannot be generalized to inform conditions in other malaria-endemic countries or other regions of Madagascar. Madagascar has a highly heterogeneous distribution of malaria burden, and each region has variable geographic, demographic, and socio-economic characteristics that can influence health-seeking behaviours [26]. As such, local circumstances even within Madagascar must be carefully examined to determine the applicability of these findings. Finally, several individuals experiencing fever noted being tested for malaria despite not seeking care from any source (see Additional file 4). This is unlikely to affect the accuracy of measurements of seeking care from CHVs or HFs, but is a noteworthy incongruity in the data collected.

## Conclusions

This survey demonstrated low reported rates of febrile illness and very low rates of associated care-seeking behaviours across all ages for febrile illness in Farafangana district. Among those with positive malaria RDTs, a minority noted preceding fever. However, 62.1% of those with febrile malaria were aged 5–14 years. This, coupled with high rates of malaria testing by CHVs, suggest that age-expanded mCCM may be an effective intervention in Farafangana to drastically increase the availability of malaria care to those in need of it.

## Supplementary Information

**Additional file 1: Figure S1**. Location and estimated accessibility of health facilities, Farafangana 2019–2020. A detailed map of the study area including estimates of duration of accessibility for study teams

**Additional file 2**: Sample size calculations. Brief description of sample size calculations for interventional trial, including assumptions used

**Additional file 3**: **Figure S2**. Prevalence of fever, care-seeking, and diagnostic testing for malaria by age group, Farafangana, Madagascar 2019. A. Population estimates of percentage of individuals noting febrile illness in preceding two weeks, by age group. Vertical lines represent 95% confidence intervals. B–D***.*** Percentage of febrile individuals seeking medical attention for fever at either HF or CHV, and percentage that were tested for malaria during their visit. Numbers to the right of braces demonstrate the percentage of those tested for malaria among only those who sought care. B***.*** Individuals under 5 years. C. Individuals 5 to 14 years. D. Individuals 15 years and older.

**Additional file 4: Figure S3**. Sankey plot of care-seeking behaviour among individuals experiencing febrile illness within two weeks prior to survey and diagnostic malaria testing at each provider. Depicted are bar charts of number of individuals who experienced a febrile illness within the two weeks prior to survey categorized by membership in different groups of (from left to right) age class, location of health care services sought, and malaria testing status. Shaded areas between bar charts represent flow of individuals from one category to another, with size of the shaded area proportional to number of individuals. Color of shading between bars correlates to an individual’s age throughout the figure. ‘Other’ category includes: self-medication (n = 8), private health facility (n = 4), marketplace (n = 2), and pharmacy (n = 1).

## Data Availability

The datasets generated and/or analysed during the current study are the property of Institut Pasteur de Madagascar and were shared with the corresponding author and CDC collaborators at the owners’ discretion. Data are not publicly available, but reasonable requests will be forwarded to the Institut Pasteur de Madagascar by the corresponding author.

## Abbreviations

CHV: Community Health Volunteer
CI: 95% Confidence interval
CU5: Children under 5 years
EA: Enumeration area
HF: Health Facility
iCCM: Integrated Community Case Management
mCCM: Malaria Community Case Management
MIS: Malaria Indicator Survey
RDT: Malaria Rapid Diagnostic Test
