# Supplementary material for: Baseline malaria prevalence and care-seeking behaviours in rural Madagascar prior to a trial to expand malaria community case management to all ages
Source: Malar J. 2021 Oct 26;20:422. doi: 10.1186/s12936-021-03956-z (PMC8549293; doi:10.1186/s12936-021-03956-z)

**Supplemental Figure 1 – Location and estimated accessibility of health facilities, Farafangana 2019­–2020**


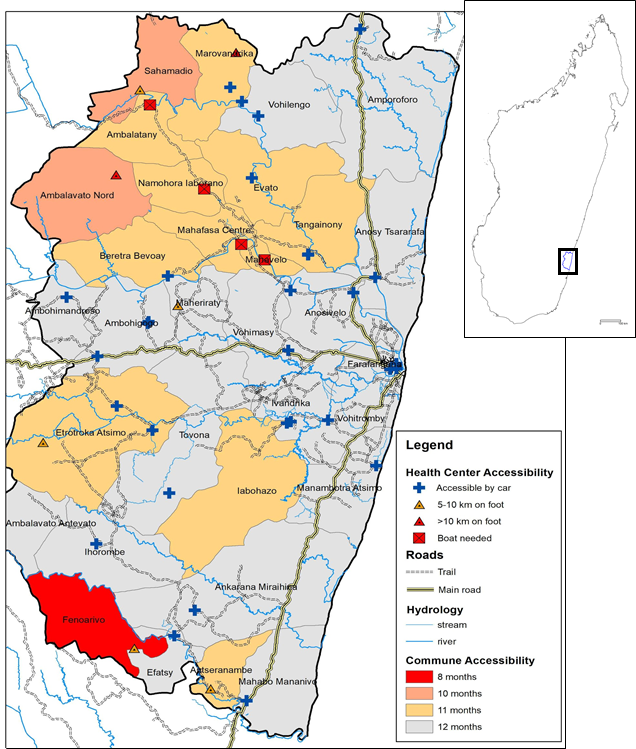

Supplement: Supplementary file 1 — Additional file 1: Figure S1. Location and estimated accessibility of health facilities, Farafangana 2019–2020. A detailed map of the study area including estimates of duration of accessibility for study teams [file 12936_2021_3956_MOESM1_ESM.docx]
