# Supplementary material for: Baseline malaria prevalence and care-seeking behaviours in rural Madagascar prior to a trial to expand malaria community case management to all ages
Source: Malar J. 2021 Oct 26;20:422. doi: 10.1186/s12936-021-03956-z (PMC8549293; doi:10.1186/s12936-021-03956-z)
Supplement: Supplementary file 2 — Additional file 2: Sample size calculations. Brief description of sample size calculations for interventional trial, including assumptions used [file 12936_2021_3956_MOESM2_ESM.docx]

Sample size calculations

Survey sample size was calculated to detect a hypothesized 17% difference between the intervention and control groups during the future end line survey for the primary endpoint of the two-year trial (proportion of individuals of all ages with febrile illness in the previous two weeks who received a blood test for malaria) with 80% power. Additional assumptions for sample size calculation included 1) 18% of those with a febrile illness at baseline would seek care, 2) 56% of households would have at least one febrile respondent, 3) non-response rate of 15%, and 4) intra-cluster correlation coefficient of 0.1.
