# Supplementary material for: Baseline malaria prevalence and care-seeking behaviours in rural Madagascar prior to a trial to expand malaria community case management to all ages
Source: Malar J. 2021 Oct 26;20:422. doi: 10.1186/s12936-021-03956-z (PMC8549293; doi:10.1186/s12936-021-03956-z)
Supplement: Supplementary file 3 — Additional file 3: Figure S2. Prevalence of fever, care-seeking, and diagnostic testing for malaria by age group, Farafangana, Madagascar 2019. A. Population estimates of percentage of individuals noting febrile illness in preceding two weeks, by age group. Vertical lines represent 95% confidence intervals. B–D. Percentage of febrile individuals seeking medical attention for fever at either HF or CHV, and percentage that were tested for malaria during their visit. Numbers to the right of braces demonstrate the percentage of those tested for malaria among only those who sought care. B. Individuals under 5 years. C. Individuals 5 to 14 years. D. Individuals 15 years and older. [file 12936_2021_3956_MOESM3_ESM.docx]

**Supplemental Figure 2. Prevalence of fever, care-seeking, and diagnostic testing for malaria by age group, Farafangana, Madagascar 2019.**


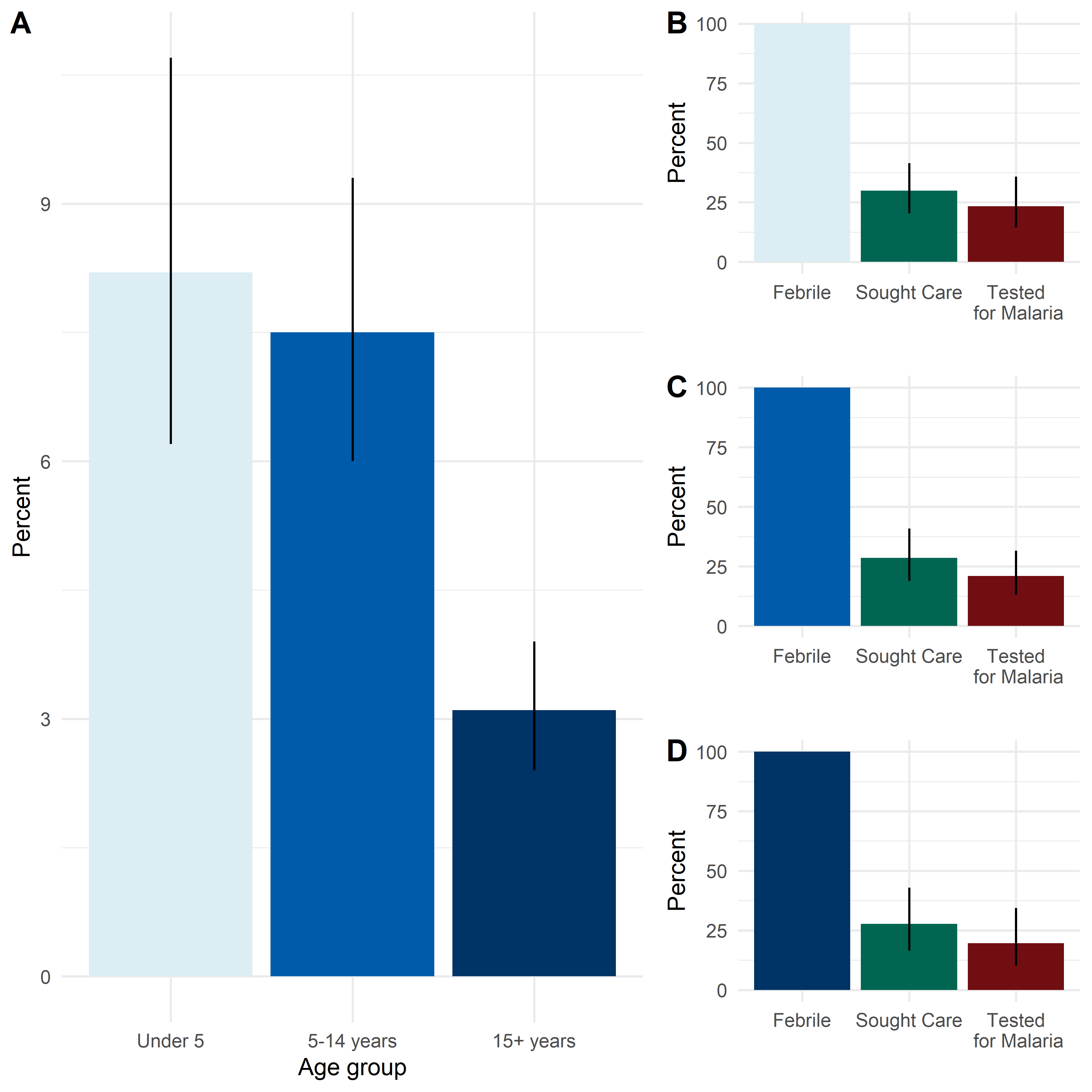


77.6%

90.3%

73.9%


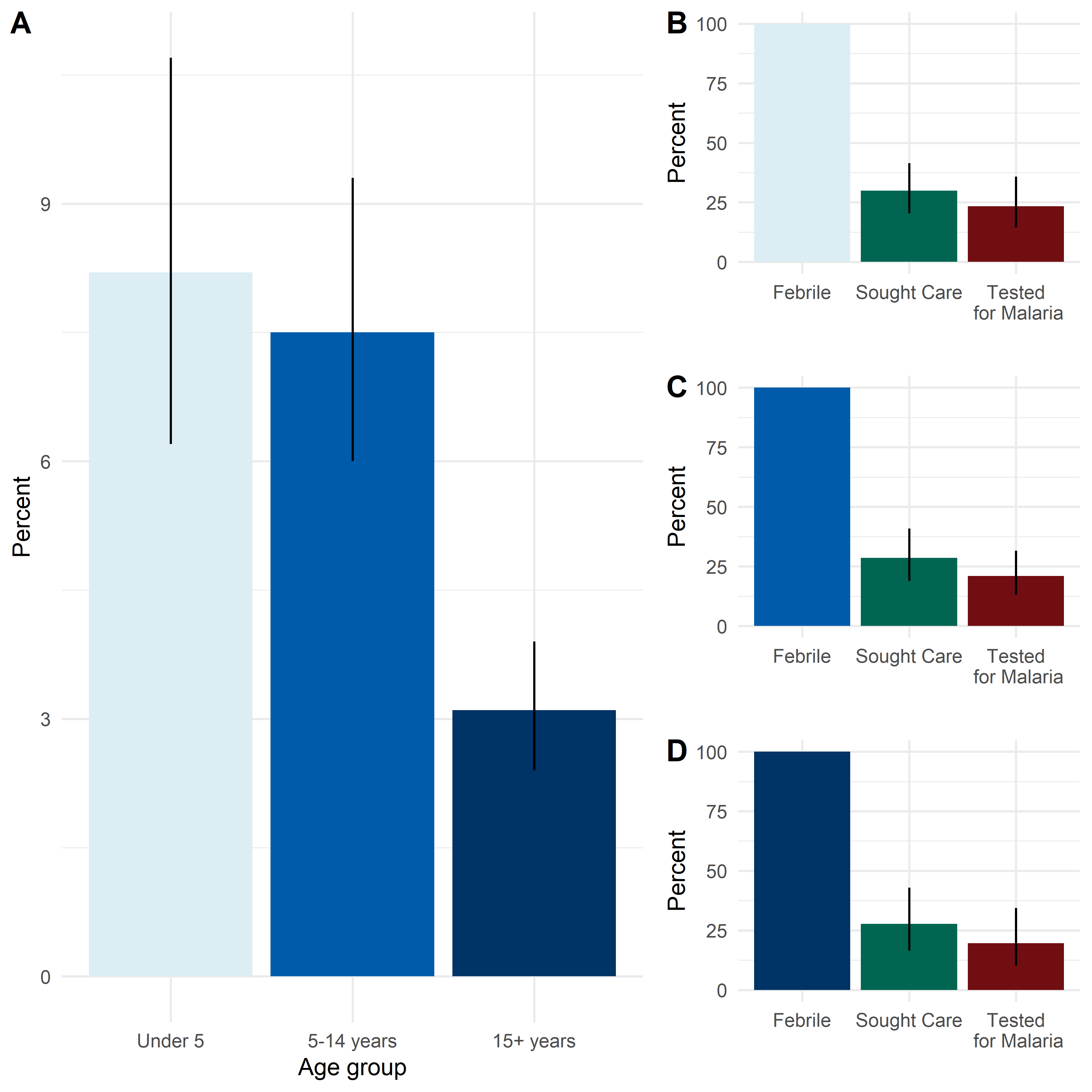


77.6%

90.3%

73.9%


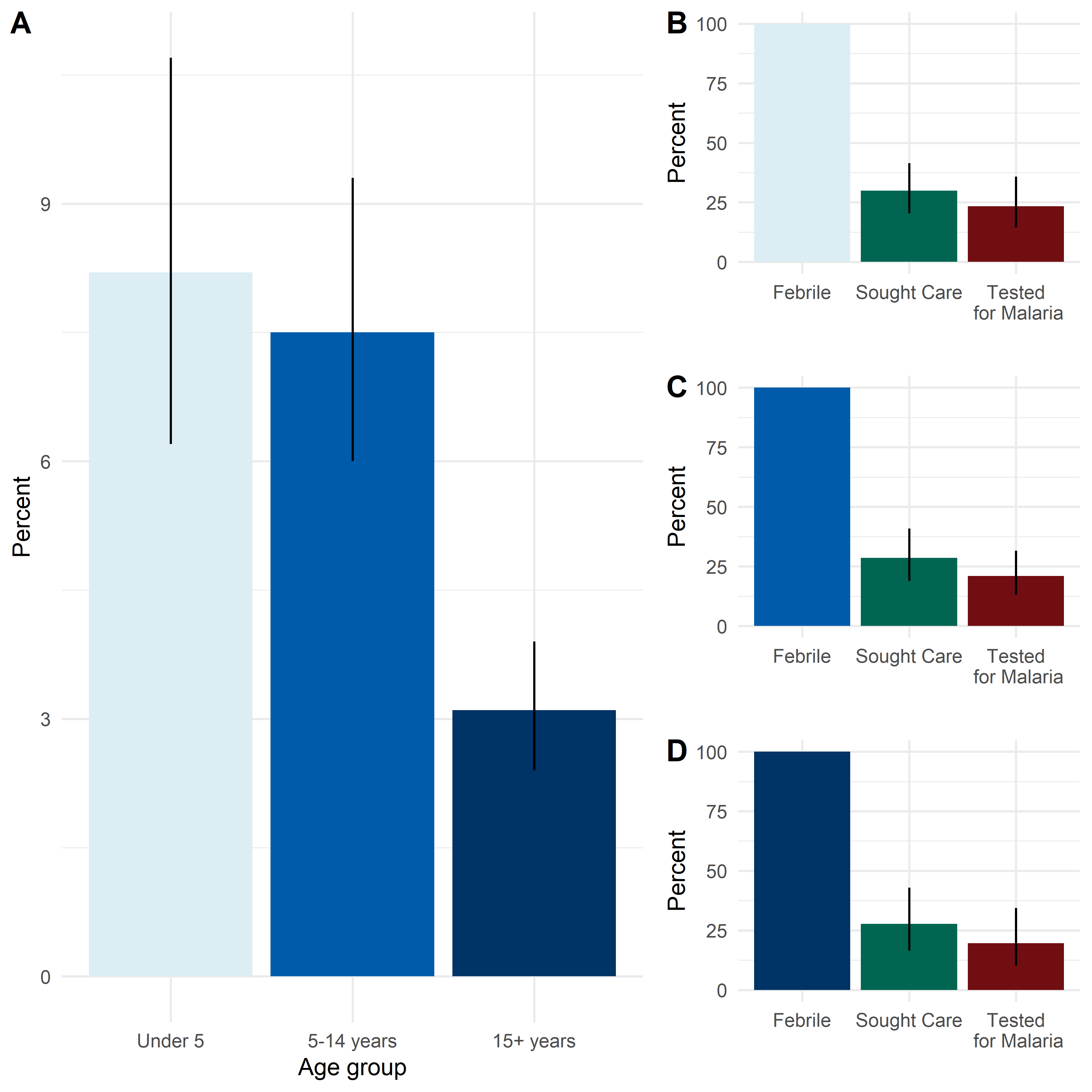


77.6%

90.3%

73.9%
