# Supplementary material for: Baseline malaria prevalence and care-seeking behaviours in rural Madagascar prior to a trial to expand malaria community case management to all ages
Source: Malar J. 2021 Oct 26;20:422. doi: 10.1186/s12936-021-03956-z (PMC8549293; doi:10.1186/s12936-021-03956-z)

**Supplemental Figure 3. Sankey plot of care seeking behavior among individuals experiencing febrile illness within two weeks prior to survey and diagnostic malaria testing at each provider.**


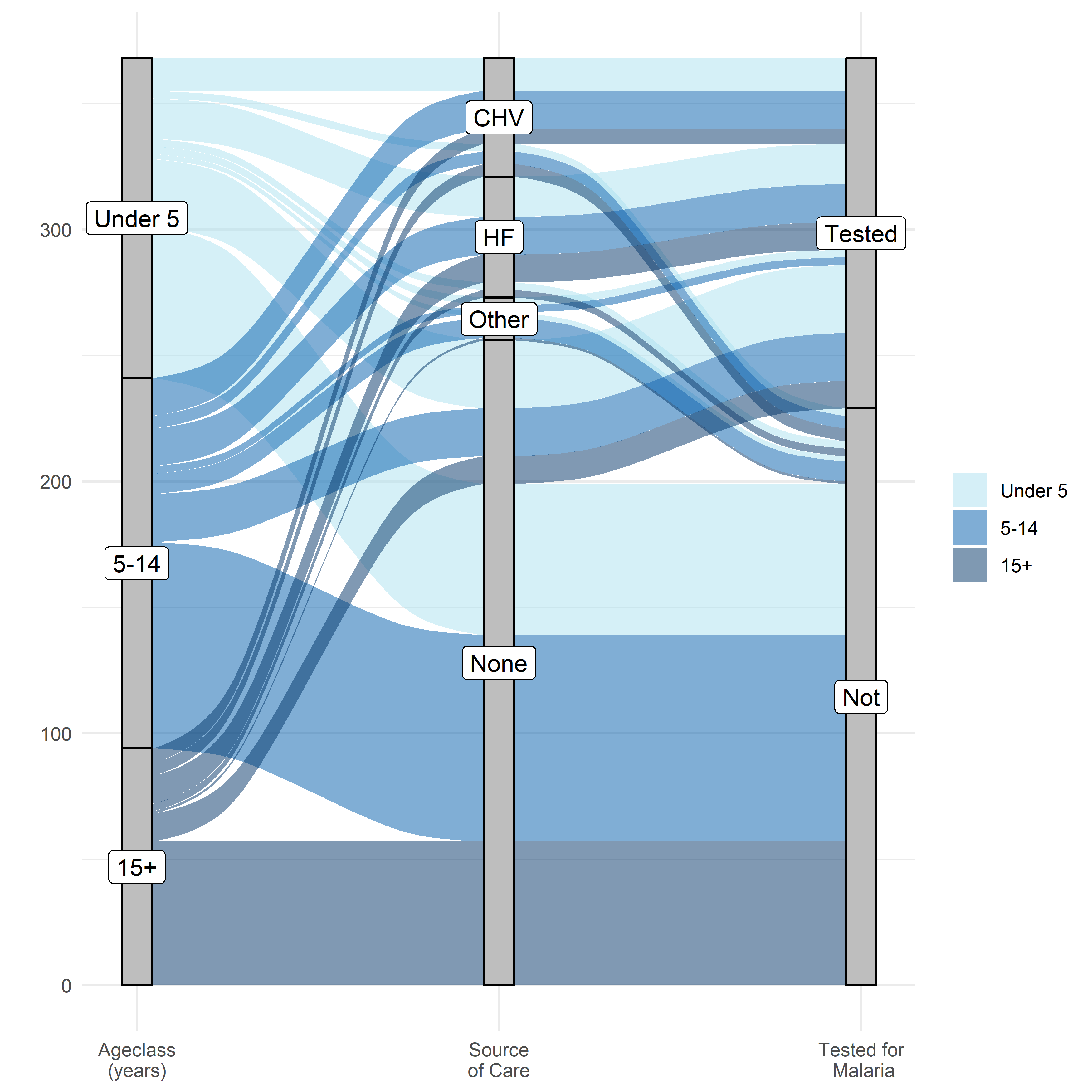

Supplement: Supplementary file 4 — Additional file 4: Figure S3. Sankey plot of care-seeking behaviour among individuals experiencing febrile illness within two weeks prior to survey and diagnostic malaria testing at each provider. Depicted are bar charts of number of individuals who experienced a febrile illness within the two weeks prior to survey categorized by membership in different groups of (from left to right) age class, location of health care services sought, and malaria testing status. Shaded areas between bar charts represent flow of individuals from one category to another, with size of the shaded area proportional to number of individuals. Color of shading between bars correlates to an individual’s age throughout the figure. ‘Other’ category includes: self-medication (n = 8), private health facility (n = 4), marketplace (n = 2), and pharmacy (n = 1). [file 12936_2021_3956_MOESM4_ESM.docx]
